# An Effective Methodology for Scoring to Assist Emergency Physicians in Identifying Overcrowding in an Academic Emergency Department in Thailand

**DOI:** 10.1186/s12911-024-02456-9

**Published:** 2024-03-21

**Authors:** Sukumpat Na Nan, Borwon Wittayachamnankul, Wachira Wongtanasarasin, Theerapon Tangsuwanaruk, Krongkarn Sutham, Orawit Thinnukool

**Affiliations:** 1 Department of Emergency Medicine, Faculty of Medicine, Chiang Mai University, 50200 Chiang Mai, Thailand; 2 Embedded System and Computational Science Lab, Chiang Mai University, 50200 Chiang Mai, Thailand

**Keywords:** Occupancy, Overcrowding, Emergency department, Perception

## Abstract

**Background:**

Emergency Department (ED) overcrowding is a global concern, with tools like NEDOCS, READI, and Work Score used as predictors. These tools aid healthcare professionals in identifying overcrowding and preventing negative patient outcomes. However, there’s no agreed-upon method to define ED overcrowding. Most studies on this topic are U.S.-based, limiting their applicability in EDs without waiting rooms or ambulance diversion roles. Additionally, the intricate calculations required for these scores, with multiple variables, make them impractical for use in developing nations.

**Objective:**

This study sought to examine the relationship between prevalent ED overcrowding scores such as EDWIN, occupancy rate, and Work Score, and a modified version of EDWIN newly introduced by the authors, in comparison to the real-time perspectives of emergency physicians. Additionally, the study explored the links between these overcrowding scores and adverse events related to ED code activations as secondary outcomes.

**Method:**

The method described in the provided text is a correlational study. The study aims to examine the relationship between various Emergency Department (ED) overcrowding scores and the real-time perceptions of emergency physicians in every two-hour period. Additionally, it seeks to explore the associations between these scores and adverse events related to ED code activations.

**Results:**

The study analyzed 459 periods, with 5.2% having Likert scores of 5–6. EDOR had the highest correlation coefficient (0.69, *p* < 0.001) and an AUC of 0.864. Only EDOR significantly correlated with adverse events (*p* = 0.033).

**Conclusion:**

EDOR shows the most robust link with ‘emergency physicians’ views on overcrowding. Additionally, elevated EDOR scores correlate with a rise in adverse events. Emergency physicians’ perceptionof overcrowding could hint at possible adverse events. Notably, all overcrowding scores have high negative predictive values, efficiently negating the likelihood of adverse incidents.

## Introduction

Emergency Department (ED) overcrowding has become a serious global issue [1, 2] Previous evidence showed that overcrowding in the ED resulted in a high mortality rate [3]. It also results in a worse quality of emergency care, including delayed administration of antibiotics in cases of sepsis [4], delays in brain imaging and rt-PA in acute stroke patients [5, 6], and decreased patient satisfaction [7, 8].

Many scores have been developed to define the crowding in the ED. Emergency De-partment Occupancy Rate (EDOR), Emergency Department Work Index (EDWIN), National Emergency Department Overcrowding Scale (NEDOCS), Real-time Emergency Analysis of Demand Indicators (READI), and Work Score have been widely used as predictors of over-crowding [9–12]. These scores were designed to assist health care professionals in detecting overcrowding situations and preventing unfavorable outcomes for emergency patients [13–15]. Despite the existence of several scores, there is still no consensus on the most appropriate method to define ED overcrowding. Most of the published studies were done in the United States, which cannot be generalized and applied in many EDs with no waiting room and role of ambulance diversion. Moreover, the complexity of score calculation with numerous requiring variables is not realistic to be used in developing countries.

Physician perception, reflecting the clinician’s sense of being busy or overwhelmed while working, is another factor considered when identifying overcrowding [16, 17]. A recent systematic study highlighted a strong connection between ED crowding and a less favorable perception of staff care, an aspect that has been relatively underexplored in the existing literature [18]. Given the inherent challenges in quantifying this subjective parameter, there has been a scarcity of data concerning the relationship between overcrowding scores and physician perception. Establishing a measure that aligns more closely with the experiences of attending emergency physicians may offer a more pragmatic tool for effectively managing a busy ED, particularly in developing nations.

This study aimed to determine the correlation between the commonly used ED crowding scores, including EDWIN, occupancy rate, and Work Score, and the modified ED-WIN, which the authors newly generated, and the real-time perceptions of emergency physicians. The secondary outcomes were the associations between the different overcrowding scores and the adverse events of the ED code activations.

## Methodology

We conducted a prospective study at Maharaj Nakorn Chiang Mai Hospital which was university hospital from Dec 24, 2019, to Jan 31, 2020. The facility is a 1,500-bed university hospital, level I trauma, and tertiary center with an ED census of about 30,000 patients per year. The ED has 16 treatment bays and monitors. The medical ED physicians include emergency medicine residents, interns, and attending emergency physicians.

The research assistants were well briefed on the study protocol and prospectively collected the data. Data included the number of physicians, the number of patients at each triage level, patients with a plan of disposition (patients waiting for admission and referral), ED code activations including of ST-Elevation Myocardial Infarction (STEMI), acute ischemic stroke, sepsis, and severe Traumatic Brain Injury (TBI), adverse events of the code activations, in-hospital cardiac arrest, and overcrowding perception of ED attending physicians. The data were collected every period, which is two hours. Therefore, we collected twelve periods per day. This is a similar method to an original EDWIN study [19]. Adverse events of the code activations include the delay of more than 90 min of wire crossing in cases of STEMI, a delay of more than 60 min of thrombolytic treatment in acute ischemic stroke, the delay of more than 60 min in the administration of antibiotics in sepsis, and the delay time of more than 60 min in surgery for severe traumatic brain injury.

All participants, attending emergency physicians or the chief emergency residents were asked to assess how overcrowded the ED was at that particular time using the six-point Likert Score: (1) not busy at all, not crowded, (2) steady, easily keeping up, (3) average: working hard, (4) more crowded and busy than desirable, (5) very busy, need external resources (doctors, nurses, ventilators or other equipment), and (6) extremely busy, hospital overcrowding code activation. All physicians were orientated, and eight different ED scenarios tested the agreement of the busy ED before participating. Interrater reliability of overcrowded ED among the physicians showed Fleiss’ Kappa of 0.582 (95% CI 0.493–0.671), *p* < 0.001 [20].

All variables were recorded by research assistants who were blinded to all physicians at that moment. “The triage system is based on Canadian Triage and Acuity Scale (CTAS). The triage score is reversed when calculating the EDWIN score, for example, the Score of 5 is for the most severe case (Level I– CTAS), and the Score of 1 is for the least (Level V– CTAS).” All identifiers were removed from the information as it was obtained.

The analysis team analyzed the overcrowding scores; EDWIN, occupancy rate, Work Score and modified EDWIN. The studied overcrowding scores were defined as follows:

1 $$ {EDWIN}^{20}= \sum {n}_{i}{t}_{i}Na(BT-BA)$$

number of patients in ED in triage category; ti is the triage category in CTAS (ordinal scale 1–5, 5 being most acute); Na is the number of attending physicians on duty at a given time; BT is the number of treatment bays, available in ED; and BA is the number of admitted patients who held in ED. For BA, the authors included the patients who were planned to be referred to another hospital and generated a new score, modified EDWIN.

The Emergency Department occupancy rate or EDOR is calculated by dividing the number of patients currently in the ED by the total number of available treatment beds in the ED. The formula is as follows:

2 $$\begin{aligned} {ED\,OR}^{12} = &Total\,Equation\,Number\, of \,patients / \\&Total\, Equation\,Number \,of \,licensed \,treatment \,bays\end{aligned}$$

In this study, the maximum number of monitored beds in the ED, which is 15, is used to calculate the EDOR score. For the Work Score, it is similar to EDWIN score in taking into account the triage level, effective ED size, and a number of providers. It divides the CTAS by the number of nurses rather than physicians. Work Score was calculated by using the following formula:

3 $$ \begin{aligned}{Work Score}^{21}=& (3.23 \times {P}_{wait}/ {B}_{T}) + (0.097 \times \\& \sum {n}_{i}{t}_{i}/{N}_{n}) + (10.92 \times {B}_{A}/{B}_{T})\end{aligned}$$

where P_wait_ is the number of patients in waiting room; B_T_ is the number of treatment bays; n_i_ is the number of patients in ED in triage category; t_i_ is the triage category in CTAS (ordinal scale 1–5, 5 being most acute); N_n_ is the number of nurses on duty at a given time; and BA is the number of admitted patients who held in ED. Thus, the primary outcome was the correlation between the ED crowding scores and the Likert Score of overcrowding reflected by the emergency physician. The secondary outcome was to identify which Score has the highest sensitivity and specificity and which relates to the code activations’ adverse events.

### Statistical analysis

The experimental size calculation was conducted based on prior statistical evidence [12]. We used the simple regression method for the primary outcome. With a power of 90% and an alpha value of 0.05, the total estimated sample size was 437. We decided to include 459 samples (5% estimation) in the data analysis in case of missing data.

The data collected for this study were organized and entered into a Microsoft Excel 2010 spreadsheet. Statistical analysis was performed using SPSS (Statistical Package for Social Sciences) version 22 (SPSS Inc., Chicago, USA). Descriptive statistics, such as median and interquartile ranges (IQR), were used for non-normally distributed variables.

Differences between the means of two groups were compared using either independent t-tests or Wilcoxon rank sum tests, depending on the data distribution. The Wilcoxon rank sum test is a non-parametric statistical hypothesis test employed to ascertain whether there exists a significant difference between the distributions of two independent samples. This test is specifically designed for situations where the assumptions of parametric tests, such as the t-test, are not satisfied, especially when dealing with ordinal or non-normally distributed data. While ANOVA was used for comparing the differences of means among more than two groups in the case of continuous data.

Categorical variables were presented as frequency and percentage and compared using a chi-square test. The correlation between crowding scores and physician perception was analyzed using the Spearman correlation coefficient.

To identify which Score shows the highest correlation with adverse events, the researchers assumed that a Likert score of 5 and 6 represents an out-of-control busyness in ED and the need for external resources as a reference standard to diagnose ED overcrowding in this study. The diagnostic incidence of adverse events was calculated using the area under the receiver operating characteristic curve (AUC). The cutoff value to predict adverse events was identified to indicate the highest sensitivity and specificity. A value of *p* < 0.05 was considered statistically significant. We presented the association of each calculated overcrowding score and the incidence of the adverse events and compared these using Chi-square tests.

## Result

During the study, 459 periods were recorded with no missing data. Twenty-four (5.2%) periods had a physician perception of a Likert score of 5 and 6 (out-of-control busyness). Four thousand two hundred thirteen patients visited the ED, of which 1,069 (25.4%) were admitted to the hospital. The characteristics, CTAS level, and the ED patients are all shown per period in Table 1. The number of physicians, nurses, and patients in the 8-hour morning and evening shifts was larger than during the night (*p* < 0.001).

**Table 1:** Characteristics of the periods included in this study

	Total	Morning shift	Evening shift	Night shift	p-value
Number of attending physicians; median (IQR)	4 (4–5)	5 (4–6)	4 (4–5)	3 (3–4)	< 0.001
Number of nurses; Median (IQR)	6 (6–6)	6 (6–6)	6 (6–6)	5 (5–5)	< 0.001
Number of patients on board; median (IQR)	9 (6–12)	11 (8–14)	11 (8–14)	6 (4–8)	< 0.001
CTAS I	1 (0–2)	1 (0–2)	1 (1–2)	1 (0–1)	< 0.001
CTAS II	2 (1–3)	3 (1–4)	3 (1–4)	1 (1–2)	< 0.001
CTAS III	4 (2–5)	5 (3–6)	4 (3–6)	3 (1–4)	< 0.001
CTAS IV	1 (0–3)	2 (1–3)	2 (1–3)	1 (0–2)	< 0.001
CTAS V	0 (0–0)	0 (0–1)	0 (0–1)	0 (0–0)	< 0.001
Number of waiting patients; median (IQR)	0 (0–0)	0 (0–0)	0 (0–0)	0 (0–0)	0.14
Number of patients with disposition plan; median (IQR)	1 (0–2)	1 (0–2)	2 (0–1)	1 (0–2)	0.01
Number of patients admitted; median (IQR)	1 (0–2)	1 (0–1)	1 (0–2)	1 (0–2)	< 0.001
Number of referral patients; median (IQR)	0 (0–0)	0 (0–0)	0 (0–0)	0 (0–0)	0.09

CTAS: Canadian Triage and Acuity Scale; IQR: Interquartile range

The average number of patients during the night shift was 6 per session (IQR, 4–8), which was lower than the morning and evening shifts (*p* < 0.001). Most patients were categorized as CTAS II and III and were more common during the morning and evening shifts compared to the night shift (*p* < 0.001). The number of patients on board varies significantly across shifts. The morning and evening shifts have a higher median of 11 patients, while the night shift has a median of 6 patients (*p* < 0.001).

Overall, code activations occurred 264 times, and the adverse events from the code activations totaled 34. Table 2 demonstrates crowding scores for different shifts in the ED. Most crowding scores are significantly higher during morning and evening shifts compared to the night shifts (*p* < 0.001), especially the EDOR. However, Work Score does not show a significant difference between shifts (*p* = 0.14). Code activations are generally higher during the morning shift compared to other shifts. Moreover, the total number of adverse events is significantly higher during the morning and evening shifts (*p* < 0.001). Conversely, the code for sepsis and the adverse events from the code sepsis mainly occurred during the evening shift. Throughout the study period, the ED experienced minimal or negligible presence of waiting patients or patients scheduled for referral.

**Table 2:** Overcrowding scores and adverse events

Crowding score; mean (SD)	Total	Morning shift	Evening shift	Night shift	p-value
EDOR	0.69 (0.08)	0.7 (0.29)	0.69 (0.25)	0.38 (0.2)	< 0.001
EDWIN	0.33 (0.04)	0.49 (0.25)	0.62 (0.24)	0.33 (0.19)	0.02
Modified EDWIN	0.40 (0.11)	0.5 (0.26)	0.56 (0.24)	0.33 (0.19)	0.02
Work score	1.15 (0.96)	1.3 (0.90)	1.63 (1.01)	1.02 (0.87)	0.14
Total Code activations (per period); Mean (SD)	0.58 (0.99)	0.95 (1.29)	0.55 (0.89)	0.20 (0.48)	< 0.001
Code stroke	0.08 (0.29)	0.13 (0.37)	0.07 (0.25)	0.04 (0.23)	< 0.001
Code STEMI	0.08 (0.30)	0.02 (0.14)	0.02 (0.14)	0	< 0.001
Code sepsis	0.37 (0.79)	0.03 (0.16)	0.04 (0.20)	0.02 (0.14)	< 0.001
Code severe Traumatic Brain Injury	0.05 (0.99)	0.01 (0.11)	0.01 (0.08)	0	0.03
Total adverse events (per period); mean (SD)	0.07 (0.28)	0.12 (0.35)	0.07 (0.28)	0.03 (0.16)	< 0.001
Code stroke	0.03 (0.17)	0.06 (0.27)	0.01 (0.08)	0.01 (0.08)	< 0.001
Code STEMI	0.01 (0.1)	0.02 (0.14)	0.02 (0.14)	0	< 0.001
Code sepsis	0.03 (0.17)	0.03 (0.16)	0.04 (0.2)	0.02 (0.14)	0.10
Code severe traumatic brain injury	0.01 (0.10)	0.01 (0.11)	0.01 (0.08)	0	0.02
In-hospital cardiac arrest	0.03 (0.20)	0.02 (0.14)	0.05(0.28)	0.02 (0.14)	< 0.001

EDOR: Emergency Department Occupancy Rate; EDWIN: Emergency Department Work Index; SD: Standard Deviation

**Fig. 1:**
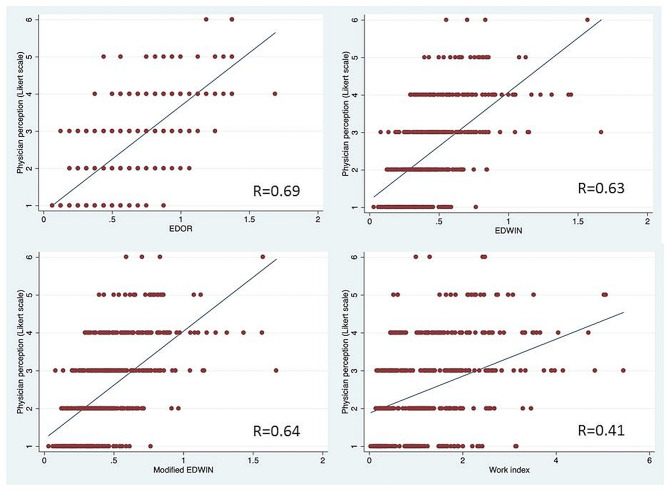
The Spearman’s correlation coefficient shows that EDOR showing the highest correlation with the physician overcrowding perception (0.69, p < 0.001), while the least correlated is Work Score (0.041, p < 0.001)

**Fig. 2:**
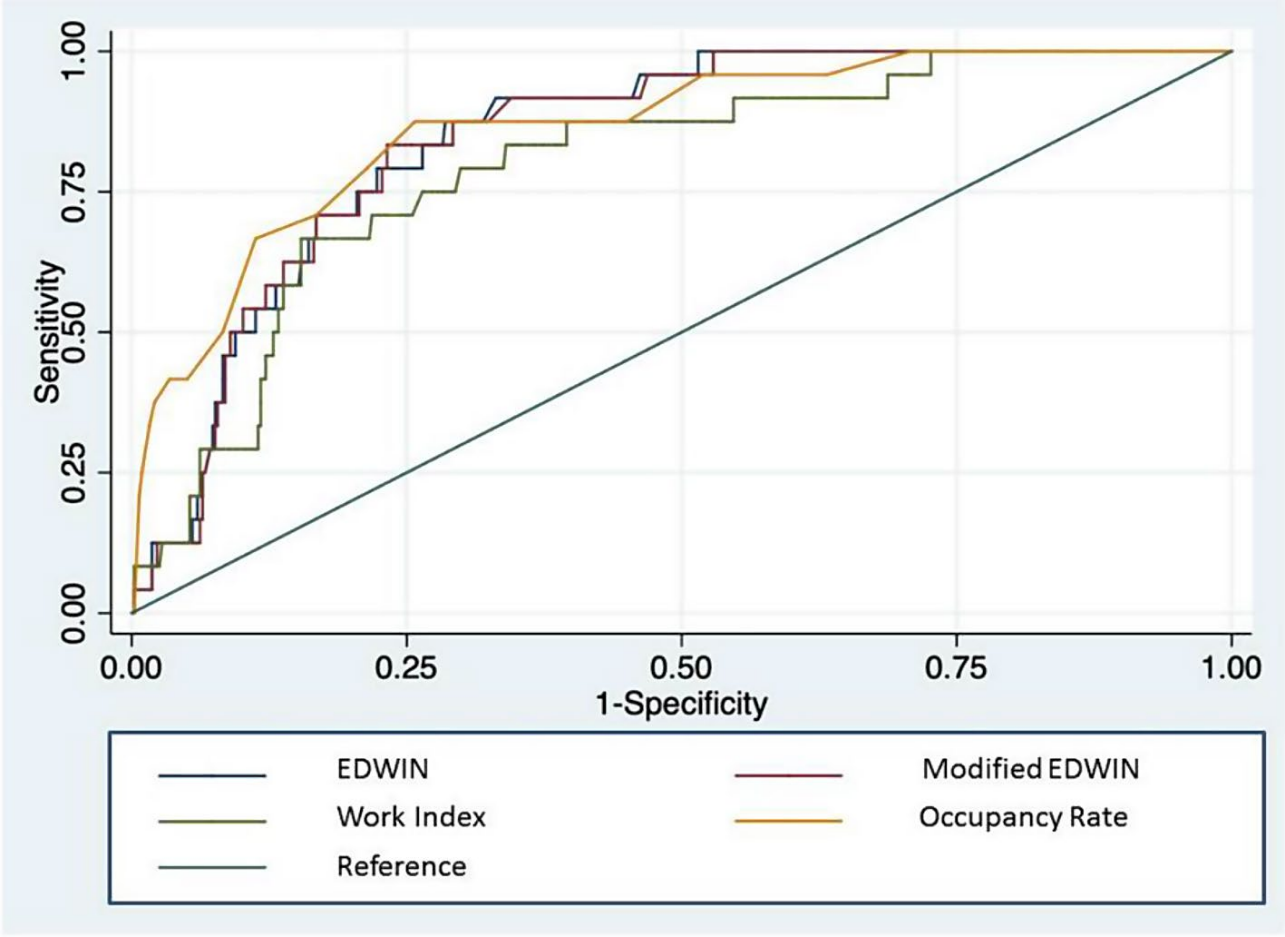
The area under the receiver operating characteristic curve (AUC) of scores to identify ED overcrowding, Occupancy Rate showing to have the greatest AUC (0.864 (95% CI 0.792–0.936, *p* < 0.001)

The Spearman’s correlation coefficient shows that EDOR shows the highest correlation with the physician overcrowding perception (0.69, *p* < 0.001), while the least correlated is work score (0.41, *p* < 0.001). (Fig. 1). Among all overcrowding scores, the AUC of scores to identify overcrowding was 0.864 (95% confidence interval [CI], 0.792–0.936, *p* < 0.001) for EDOR, 0.846 (95% CI, 0.788–0.903, *p* < 0.001) for EDWIN and 0.846 (95% CI, 0.787–0.904, *p* < 0.001) for modified EDWIN and 0.795 (95% CI, 0.713–0.878), *p* < 0.001) for Work Score (Fig. 2).

An EDOR cut-off value of 0.78 has a sensitivity, specificity, PPV, and NPV of 40.6%, 76.8%, 11.6%, and 94.5%, respectively. By using this value, 112 (24.4%) periods were identified as overcrowded. EDOR is the only Score that shows a significant correlation with adverse events from the code activations (*p* = 0.033, Table 3).

**Table 3:** Specificity, PPV, and NPV of all overcrowding scores for total adverse events from code activations

Overcrowding scores	Cut point	Overcrowding; Period (%)	Sensitivity; % (95% CI)	Specificity; %(95% CI)	PPV; %(95% CI)	NPV; %(95% CI)	p-value
EDOR	0.781	112 (24.4)	40.6 (23.7–59.4)	76.8 (72.5–80.7)	11.6 (6.3–19.0)	94.5 (1.6–96.7)	0.033
EDWIN	0.582	117 (25.5)	28.1 (13.7–46.7)	74.7 (70.3–77.8)	7.7 (3.6–14.1)	93.3 (90.1–95.7)	0.679
Modified EDWIN	0.590	118 (25.7)	34.4 (18.6–53.2)	74.9 (70.5–79.0)	9.3 (4.8–16.1)	93.8 (90.7–96.1)	0.293
Work Score	1.651	149 (32.5)	40.6 (23.7–59.4)	68.1 (63.5–72.5)	8.7 (4.7–14.5)	93.9 (90.6–96.3)	0.330

EDOR: Emergency Department Occupancy Rate; EDWIN: Emergency Department Work Index; NPV: Negative Predictive Value; PPV: Positive Predictive Value; CI: Confidence Interval

## Discussion

This study found that the score showing the most significant correlation between ED over-crowding and physician perception is EDOR. The ED overcrowding was defined as the attending physician feeling that ED crowding at that moment ranged from “very busy; need external resources” to “extremely busy; need hospital overcrowding code activation.” Despite the EDOR bearing the closest relationship to the perception of emergency physicians to over-crowding, the fit between the estimation of EDOR and ED emergency physician crowding perceptions shared the same performance as other scores. The main idea behind our study’s crowding score equations is the number of workloads divided by the number of resources. The superiority of EDOR could probably be explained by the weighting of patient severity in EDWIN, which we found too coarse a measure. A score of 5 may not be suitable for the most severe patients, and a score of 1 showed too little discrimination. Some previous studies found that the widely used EDWIN and NEDOCS are strongly associated with healthcare providers’ perceptions [19, 21]. However, EDOR is non-complicated and much easier to calculate and communicate among those scores. On the contrary, there was poor agreement be-tween the READI score and staff perception of crowding [16].

The EDOR was shown to have the greatest AUC. Previous studies showed similar re-sults [12, 22–24]. We found that EDOR is the easiest and simplest score and confers an advantage in estimating ED crowding over more complex multidimensional scores. Consequently, it lends itself readily to real-time monitoring, providing insights into the extent of overcrowding in theEDas perceived by attending physicians who require extra assistance. The simpler the measurement tool, the higher the likelihood of alleviating overcrowding and reducing the occurrence of adverse events in the ED.

This study is the first to demonstrate a significant association between higher EDOR and increased adverse events resulting from code activations. Furthermore, all overcrowding scores exhibited a high negative predictive value. Consequently, when the score falls below the established cutoff point, the likelihood of an adverse event is low. EDOR has the potential to predict the overall occurrence of adverse events related to code activations. However, given the low incidence of adverse events ob-served in this study, the EDOR was not able to independently predict the occurrence of adverse events.

Similar to the findings of a previous study [23], the high levels of overcrowding were in the morning and evening shifts, but rarely at night, despite the number of medical personnel being lower in the night shift. In addition, the number of adverse events did not increase.

The incidence of in-hospital cardiac arrest (IHCA) in this study is very low; therefore, we cannot conclude that overcrowding is associated with IHCA. IHCA is perceived as one of the complex adverse events that might not be explained by a single component, especially regarding ED overcrowding. However, the evidence regarding the association between IHCA and ED overcrowding is still limited. Two studies conducted in Korea [25, 26] showed a significant correlation between these two parameters, whereas Chang [27] found no relationship.

In this study, we designed a ‘modified EDWIN’ score by adding the planned referral patients into the original EDWIN score and assessed the accuracy. We formulated a ‘modified EDWIN’ score by incorporating planned referral patients into the original EDWIN score and evaluated its precision. Nonetheless, we encountered a significant limitation due to the remarkably low number of referred patients observed during the study, rendering the validation of this novel score inadequate.

This study has some important implications. The real-time EDOR was found to be beneficial for the timely management of overcrowding in Emergency Departments to reduce unfavorable outcomes. When a physician perceives being overcrowded and needing more resources, EDOR can be an accurate indicator representing those subjective feelings and ad-dressing the high-risk situation of ED for an adverse event. Implementing interventions to ensure that the EDOR remains below the cutoff point may lead to a reduced likelihood of adverse events. Additionally, our study assessed real-time physician perceptions of ED overcrowding and discovered a correlation with over-crowding scores. Our findings underscore the potential value of physician perception as a dependable indicator for identifying ED overcrowding.

## Conclusion

In this study, the EDOR demonstrates the strongest correlation with emergency physicians’ perception of overcrowding. Furthermore, higher EDOR scores are also associated with an increased incidence of adverse events. Physicians’ perception of overcrowding may serve as an indicator for potential adverse events. Importantly, all overcrowding scores exhibit high negative predictive values, effectively ruling out the occurrence of adverse events.

## Limitations

There are some limitations to our study. Firstly, the study was conducted at a single tertiary center. Clinical practice guidelines may vary among other hospitals, potentially impacting ED crowding throughput and outcomes. Generalizing findings to other EDs would be difficult. Secondly, there was insufficient evidence regarding adverse events during the overall sample periods. Conducting a subsequent study specifically focused on adverse events related to code activations would help address these issues.

Thirdly, the overcrowding scores in this study measured different aspects of ED situations, aiming insights into potential solutions. However, comparing their sensitivity and specificity is challenging due to their specificity to a particular variable of hospital’s ED. Moreover, other studied ED crowding scores were not included (NEDOCS, READI, International Crowding Measure in Emergency Departments or ICMED, Community Emergency Department Overcrowding Score or CEDOCS, and Severely-overcrowding Overcrowding and Not-overcrowding Estimation Tool or SONET). Some variables are unable to obtain in our setting due to the lack of a separate waiting room. However, we designed to study the common and non-complex scores, which may easily apply to most hospitals in upper-middle-income countries. The lack of waiting facilities could affect the accuracy of the waiting patient number. We also found that the number of patients planned for referral was too small to show any difference.

In the further research, an innovative approach could involve developing a new measurement that combines these tools, especially with data from multiple hospital EDs. It also should be conducted during different seasons. This study took place in winter, and it’s possible that overcrowding might be a more significant issue in summer, especially concerning conditions like sepsis. Lastly, our study did not elucidate the reasons behind each overcrowding period; additional research should aim to identify the causes or consequences of ED overcrowding.

## Data Availability

The datasets used and/or analysed during the current study are available from the corresponding author on reasonable request. The data confidentiality of information was maintained anonymously. All methods were carried out in accordance with relevant guidelines and regulations. Moreover, informed consent was employed to respondents by explaining the purpose of the study as well as maintaining the subjects’ confidentiality.

## Abbreviations

ED: Emergency department
NEDOCS: National Emergency Department OverCrowding Score
READI: Real-time Emergency Analysis of Demand Indicators
EDWIN: Emergency Department Work Index
EDOR: Emergency Department Occupancy Rate
STEMI: ST-Elevation Myocardial Infarction
TBI: Traumatic Brain Injury
CTAS: Canadian Triage and Acuity Scale
SPSS: Statistical Package for Social Sciences
IQR: interquartile ranges
ANOVA: Analysis of Variance
AUC: Area Under the Curve
